# Profile of brazilian research productivity grant holders with a background in pediatric dentistry

**DOI:** 10.1590/0103-6440202205016

**Published:** 2022-10-21

**Authors:** Larissa Chaves Morais de Lima, Veruska Medeiros Martins Bernardino, Isolda Mirelle de Lima Ferreira Prata, Roanny Torres Lopes, Samara Ellen da Silva, Myrelle Leal Campos Sousa, Matheus França Perazzo, Saul Martins Paiva, Ana Flavia Graville-Garcia

**Affiliations:** 1 Post-Graduation Program in Dentistry, Dental School, Universidade Estadual da Paraíba - UEPB, Campina Grande, PB, Brazil; 2 Graduation in Dentistry, Dental School, Universidade Estadual da Paraíba - UEPB, Campina Grande, PB, Brazil; 3 Department of Oral Health, Dental School, Federal University of Goiás - UFG, Goiânia, GO, Brazil; 4 Department of Pediatric Dentistry and Orthodontics, Dental School, Universidade Federal de Minas Gerais - UFMG, Belo Horizonte, MG, Brazil

**Keywords:** researcher, pediatric dentistry, research funding

## Abstract

This study outlines the profile of research productivity grant holders of the *Conselho Nacional de Desenvolvimento Científico e Tecnológico* [CNPq (National Council for Scientific and Technological Development)] in the field of pediatric dentistry. A cross-sectional study with data collected from the Brazilian academic curriculum vitae database. The eligibility criterion was being a research productivity grant holder in pediatric dentistry from 2018 to 2020. In the period of interest, 215 individuals were research productivity grant holders in the field of dentistry, 33 of whom had graduate degrees (specialization, master's or doctorate) in pediatric dentistry. The period of scientific production and work concluded of advising of scientific initiation, master, doctoral and post-doctoral degrees was 2010 to 2020. Descriptive analysis was performed and the Kruskal-Wallis test was used to analyze associations (5% significance level) between productivity grant level (2, 1D, 1C, 1B or 1A) and year of obtainment of the doctoral degree. The VOSviewer (version 1.6.17) was used to present graphically the interinstitutional collaborations. The sample was composed of Level 2 researchers (66.7%), women (66.7%), researchers linked to institutions in the southeastern region of Brazil (81.8%), with a doctoral degree concluded prior to 2002 (51.5%), began working as a professor at a higher education institution prior to 2007 (78.8%) and the title of full professor (45.5%). No significant association was found between productivity grant level and year of conclusion of the doctoral degree (p = 0.10). Median (interquartile range) of scientific articles was 119 (37-312). The prevalence of citations (57.52%) and JCR articles (62.76%) was higher among female researchers. In conclusion, CNPq research productivity grant holders in pediatric dentistry are essentially represented by females from the southeast region of the country (UFMG and USP). However, males have proportionally greater productivity.

## Resumo

Este estudo traça o perfil dos pesquisadores bolsistas de produtividade do Conselho Nacional de Desenvolvimento Científico e Tecnológico (CNPq) da área de odontopediatria. Pesquisa transversal com dados coletados na base de currículos acadêmicos brasileiros. Os critérios de elegibilidade abrangeram os pesquisadores bolsistas de produtividade de 2018 a 2020. Foram identificados 215 pesquisadores bolsistas de produtividade na área de odontologia, dos quais 33 com pós-graduação (especialização, mestrado ou doutorado) em odontopediatria. O período de produção científica e trabalho de orientação concluído de iniciação científica, mestrado, doutorado e pós-doutorado foi de 2010 a 2020. Foi realizada análise descritiva e o teste de Kruskall-Wallis foi utilizado para verificar associações (nível de significância de 5%) entre nível da bolsa de produtividade (2, 1D, 1C, 1B ou 1A) e ano de obtenção do grau de doutor. O programa VOSviewer (versão 1.6.17) foi utilizado para identificar colaborações interinstitucionais. A amostra foi composta por pesquisadores de Nível 2 (66,7%), mulheres (66,7%), pesquisadores vinculados a instituições da região sudeste do Brasil (81,8%), com doutorado concluído antes de 2002 (51,5%), começaram a atuar como professor em instituição de ensino superior anterior a 2007 (78,8%) e o título de professor titular (45,5%). Não foi encontrada associação significativa entre nível de bolsa de produtividade e ano de conclusão do doutorado (p = 0,10). A mediana (intervalo interquartil) de artigos científicos foi de 119 (37-312). A prevalência de citações (57,52%) e artigos do JCR (62,76%) foi maior entre as pesquisadoras. Em conclusão, os pesquisadores bolsistas de produtividade em Odontopediatria do CNPq são representados essencialmente por mulheres da região sudeste do país. No entanto, os homens apresentaram produtividade proporcionalmente maior.

## Introduction

Scientific knowledge is the driving instrument of the socioeconomic development of a country and helps improve the quality of life of the population. Such knowledge is built through investigations conducted in the academic environment ^1^. Partnerships among universities, private enterprises and governments seek ways to minimize social problems as well as promote scientific and technological innovation ^2^. This scenario also places demands on students and professors of graduate courses to produce a quantity of scientific publications ^3^.

The *Conselho Nacional de Desenvolvimento Científico e Tecnológico* (CNPq [National Council for Scientific and Technological Development] is a Brazilian fostering agency that provides productivity grants to researchers linked to graduate courses. Researchers are classified as level 1, level 2 and senior researcher. The time in which the researcher concluded the doctoral degree is the main requirement for the classification. Level 1 researchers are also further classified (A, B, C or D) based on preestablished criteria, such as scientific production of quality, especially in their field of expertise ^4^ ^,^ ^5^.

The Brazilian government increased funding to research in the health field by 325% between 2003 and 2010. This period also corresponded to considerable financial incentives in the career of researchers affiliated with public universities and was related to a 145% increase in the publication of scientific articles in different fields of knowledge, including dentistry ^3^. This situation contributed to the establishment of graduate courses in dentistry in recent years.

However, the quantity of articles published does not always mean excellence in scientific production and, consequently, may not result in improvements in the quality of life of individuals ^6^, despite being a factor that influences the obtainment of a research grant ^7^. Quality is demonstrated when an article is cited in other studies and is used as the basis for investigations or discussions ^8^. Thus, the Coordenação de Aperfeiçoamento de Pessoal de Nível Superior (CAPES [Coordination for the Advancement of Higher Education Personnel]) began to demand of professors affiliated with graduate courses a greater quantity of scientific production in periodicals with a high "QUALIS" rating, such as international journals of greater impact ^5^.

 This periodic evaluation of professors and graduate courses by CAPES led to the search for greater educational qualification as well as partnerships with other graduate programs for the development of research with greater scientific impact and social relevance. As a result, the production of new knowledge as well as scientific and technological innovation elevated Brazil's position in the world research scenario ^9^.

Thus, Brazilian dentistry was lifted to a prominent position on the international stage, accounting for about 7% of all scientific production in recent years. The 16,333 articles published between 1996 and 2015 elevated Brazil to the second country with the largest number of scientific publications, surpassed only by the United States of America (USA) - a difference that has been diminishing every year. This is considered a recent phenomenon initiated in this century, during which Brazilian dentistry has had one of the largest average increases in the number of scientific publications. The fields of pediatric dentistry and oral health in childhood and adolescence have had greatest adherence to the agenda, followed by collective health, collective oral health, dental materials, prostheses and endodontics ^1^.

Several scientific investigations have analyzed the profile of researchers with CNPq research productivity grants in different fields of knowledge ^6^ ^,^ ^10^. Although pediatric dentistry is one of the fields of dentistry that has contributed to significant scientific production and is more in line with the oral health policy of the country ^1^, no studies have evaluated the profile of researchers in pediatric dentistry who hold scientific productivity grants. Therefore, the aim of the present study was to evaluate the profile of CNPq research productivity grant holders in the field of pediatric dentistry in the period from 2018 to 2020.

## Methods

A descriptive, analytical, cross-sectional study was conducted. A list was obtained of all researchers receiving CNPq grants for active research in the field of dentistry in the three-year period from 2018 to 2020. A total of 215 grant holders were identified and 33 with a graduate degree (specialization, master's or doctorate) in pediatric dentistry were selected for the present study. The curriculum vitae (CV) of each selected researcher was consulted though the CNPp Lattes platform (https://lattes.cnpq.br/).

Access to the CVs enabled the collection of information and creation of a databank. Spreadsheets were created with information on the researcher's sex, region of the affiliated institution, researcher's title at the higher education institution, the total number of articles published and the total number of articles published in journals indexed in Journal Citation Reports (JCR). The period of scientific production and work concluded in terms of advising students of scientific initiation, master, doctoral and post-doctoral program was 2010 to 2020. Data were also collected on the year of obtainment of the doctoral degree, the year in which the researcher began working at the higher education institution (considering the median) and the level of the research productivity grant (2, 1D, 1C, 1B or 1A).

The three main titles for higher education professors in Brazil are Associate, Adjunct and Full Professor. The Ministry of Education (MEC) has broad, robust criteria for the progression of staff members in each category, which is generally related to time, performance and production during academic life. The initial category for all higher education professors is Associate. The professor must perform activities in postgraduate courses, advise students and coordinate research projects in order to be able to progress to the Assistant category. It is essential to show a clear, solid line of research in this phase. Full Professors are those who have been in the teaching career for the longest time and, in addition to performing the duties of the previous classes, coordinate research and have a greater number of scientific productions ^11^.

Regarding scientific production, data were collected on the total number of scientific articles published in journals indexed in national and international bibliographic databases as well as books and book chapters. The number of citations and self-citations of each researcher were collected during a search conducted in the Web of Science platform. Descriptive analysis of the data was performed. The non-parametric Kruskal-Wallis test was used to analyze associations (5% significance level) between productivity grant level and year of obtainment of the doctoral degree as well as the grade of the graduate program to which the researcher was affiliated. The statistical analysis was performed with the aid the SPSS Statistics for Windows (version 25).

VOSviewer version 1.6.17 was used to identify partnerships among researchers affiliated with different higher education institutions in Brazil with shared authorship in scientific production. The nominal presentation of each author was not performed in order to avoid direct comparisons between researchers. There was only one UNESP campus (Araçatuba, SP) due to the fact that only one researcher was a CNPq grant holder. The VOSviewer program enables the construction and visualization of bibliometric maps and is available free of charge to the scientific community at the website www.vosviewer.com. The names of the universities were identified with different colors and circle size was positively correlated with more scientific production and collaborations in authorships. The VOSviewer program was used to create bibliometric networks. Institutions were organized into clusters, with each cluster represented by a color. More important terms have larger circles and strongly related terms are closer to each other. Lines between institutions indicate relations and thicker lines represent a stronger link between institutions ^12^.

## Results

Table 1 displays the characterization of the researchers in pediatric dentistry who received productivity grants from CNPq in the study period. Women (66.7%) and residents of the southeastern region of Brazil (81.8%) predominated in the sample. Most researchers (51.5%) obtained their doctoral degree by 2002 and began working at a higher education institution by 2007 (78.8%), at which they maintained their current title of full professor (48.5%). Level 2 researchers accounted for the largest portion of the sample (n = 22; 66%) and published more articles. On all productivity grant levels, more than half of the articles were published in JCR periodicals.

**Table 1:** Characterization of researchers in pediatric dentistry who received cnpq productivity grants in the study period.

	n (%)	Grant Level					
		Level 2	Level 1D	Level 1C	Level 1B	Level 1A	Senior researcher
Sex							
Female	22 (66.7)	15	2	3	1	1	-
Male	11 (33.3)	7	1	-	2	1	-
Region							
North	0 (0.0)	-	-	-	-	-	-
Northeast	3 (9.1)	3	-	-	-	-	-
Central West	1 (3.0)	1	-	-	-	-	-
Southeast	27 (81.8)	16	3	3	3	2	-
South	2 (6.1)	2	-	-	-	-	-
Obtainment of doctoral degree							
Up to 2002	17 (51.5)	8	3	2	2	2	-
After 2002	16 (48.5)	14	-	1	1	-	-
Began working at higher education institution							
Up to 2007	26 (78.8)	9	2	2	2	2	-
Ager 2007	07 (21.2)	13	1	1	1	-	-
University title							
Adjunct	3 (9.1)	3	-	-	-	-	-
Associate	14 (42.4)	12	-	1	1	-	-
Full	16 (48.5)	7	3	2	2	2	-
Articles published							
Total	4380	2674	504	354	399	449	-
JCR	2624	1437	267	236	225	293	

Table 2 displays the scientific productivity of the overall sample of researchers in pediatric dentistry and according to grant level. Greater scientific productivity corresponded to scientific articles, with a median (interquartile range) of 119 (37-312), followed by book chapters (median: 2 [0-71]) and books (median: 1 [0-28]). The medians among Levels 2, 1D, 1C, 1B and 1A researchers were 119.05 (37-297), 201 (56-247), 119 (104-131), 140 (112-147) and 224.5 (137-312), respectively. On Levels 1A and 1B, the medians of advisement of doctoral students were higher than medians of advisement of master's students: 19 (17-22) and 10 (9-12), respectively.

**Table 2:** Scientific productivity of overall sample and according to cnpq productivity grant level.

	Overall (n = 33)		Grant Level									
			Level 2 (n=22)		Level 1D (n = 3)		Level 1C (n = 3)		Level 1B (n = 3)		Level 1A (n = 2)	
	Median	Range	Median	Range	Median	Range	Median	Range	Median	Range	Median	Range
Intellectual Production												
Articles	119	37-312	119.05	37-297	201	81-247	119	104-131	140	112-147	224.5	137-312
Books	1	0-28	3	0-19	0.0	0-1	0	0-0	4	0-28	6.5	1-12
Book chapters	2	0-71	1	0-71	0	0-21	0	0-3	2	0-42	16	3-29
Advising Capacity												
Scientific initiation	12	0-41	14.5	0-36	9	9-29	5	4-11	17	1-41	13	8-18
Master’s	9	2-36	8.5	4-36	12	8-12	10	4-18	3	2-8	11	7-15
Doctorate	8	0-22	6	0-15	9	8-16	9	2-20	10	9-12	19	17-22
Post-Doctorate	1	0-15	0	0-11	1	0-8	5	4-6	0	0-0	6.5	0-15

Table 3 displays the distribution (proportional to the sex of the researcher) of total citations, total citations minus self-citations, total number of articles and total number of JCR articles according to productivity grant level and sex. The ratio between the total number and quantity of researchers of each sex revealed that male researchers had more citations and articles published compared to female researchers and these differences were greater on Levels 1A and 1B. Moreover, when analyzing citations with and without self-citations, the number of self-citations did not exert a significant impact on the ratio per productivity level. 

No statistically significant association was found between productivity grant level and year of doctoral degree obtainment or grade of graduate program (p > 0.05) (Kruskal-Wallis test).

**Figure 1:**
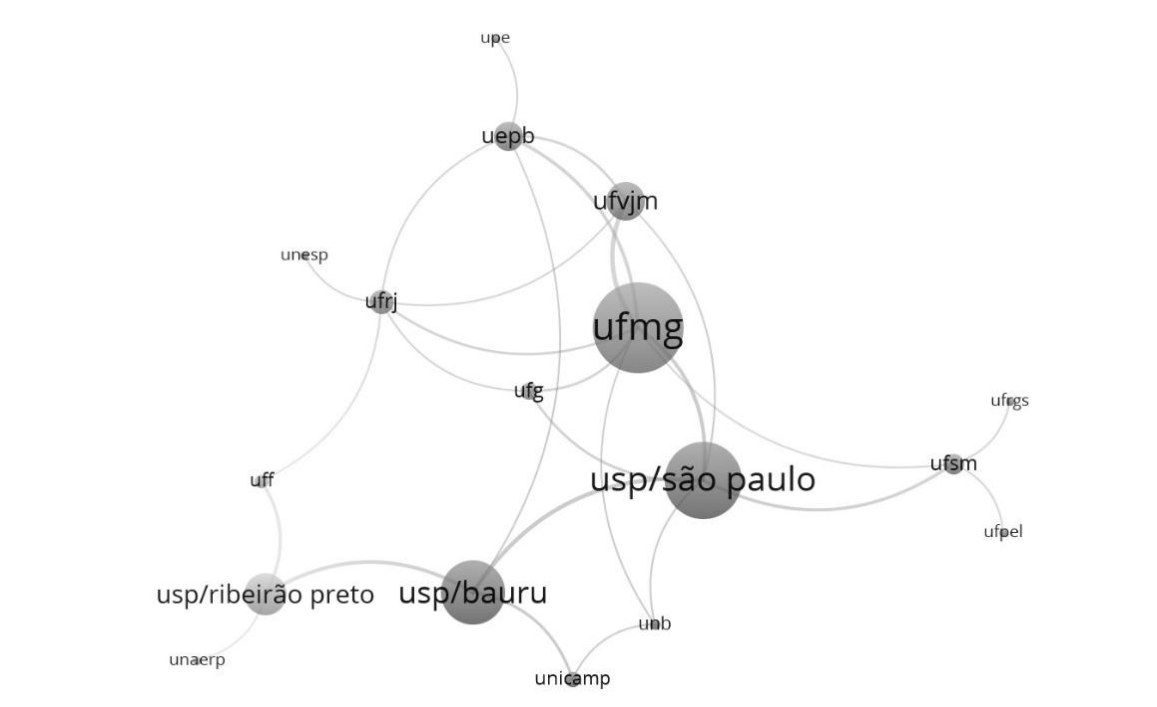
Infographic illustration of interinstitutional collaboration among CNPq productivity grant holder in pediatric dentistry in different regions of Brazil.

Figure 1 displays collaborations among productivity grant holders at different higher education institutions in Brazil. Collaborations in the publication of scientific articles were found among all researchers, with a noteworthy contribution of institutions in the southeast region of the country. More publications and collaborations were found for researchers of Universidade Federal de Minas Gerais (UFMG) and Universidade de São Paulo (USP) in the cities of São Paulo, Bauru and Ribeirão Preto.

**Table 3:** Distribution of total citations, total citations minus self-citations, total number of articles and total number of jcr articles according to productivity grant level and sex.

	Sex of Researcher			
	Female		Male	
	n (%)	Ratio*	n (%)	Ratio*
Citations				
Total citations	20576 (57.5)	935.3	15193 (42.5)	1381.2
Level 2	11884 (33.3)	792.26	6610 (18.5)	600.9
Level 1D	2868 (8.0)	1434	1424 (3.9)	1424
Level 1C	3436 (9.6)	114.5	-	-
Level 1B	764 (2.1)	764	4805 (13.4)	2402.5
Level 1A	1624 (4.5)	1624	2354 (6.7)	2354
Without self-citations	18702 (57.5)	850.1	13079 (42.5)	1189
Level 2	10612 (33.4)	707.5	6054 (19.0)	864.85
Level 1D	2623 (8.3)	1311.5	1188 (3.7)	1188
Level 1C	3215 (10.1)	1071.6	-	-
Level 1B	700 (2.2)	700	3803 (12.0)	1901.5
Level 1A	1552 (4.9)	1552	2034 (6.4)	2034
Articles				
Total articles	2787 (63.6)	126.7	1593 (36.4)	144.8
Level 2	1828 (41.7)	83.1	821 (18.7)	117.3
Level 1D	328 (7.5)	164	201 (4.6)	201
Level 1C	354 (8.1)	118	-	-
Level 1B	140 (3.2)	140	259 (5.9)	129.5
Level 1A	137 (3.1)	137	312 (7.1)	312
JCR articles	1647 (62.7)	74.8	977 (37.3)	88.8
Level 2	1057(40.3)	48.04	446 (17.0)	63.7
Level 1D	167(6.4)	83.5	100 (3.8)	100
Level 1C	236(9.0)	78.6	-	-
Level 1B	97(3.7)	97	228 (8.7)	114
Level 1A	90(3.4)	90	203(7.7)	203

*Ratio between total number and quantity of researchers of each sex.

## Discussion

The present analytical cross-sectional study outlined the profile of researchers in the field of pediatric dentistry who received scientific productivity grants from CNPq [National Council for Scientific and Technological Development] in the period from 2018 to 2020. The CNPq productivity grant constitutes a milestone in a researcher's academic career in dentistry. Dozens of researchers compete for grants on different levels. Level 2 is the first CNPq grant level and it is only possible to obtain this grant if another researcher is not able to maintain his or her level or proceeds to a higher level. CNPq does not offer new fellowships very often. Being selected for level 1A is more restricted and requires greater academic qualifications ^5^. Knowledge regarding the profile of these researchers in pediatric dentistry is important to all those who wish to achieve this benchmark in their careers and for recent doctors who wish to progress in research activity.

In the present study, the most prevalent level was Level 2 and none of the researchers analyzed achieved the level of Senior Researcher. Similar findings were reported for researchers in the field of dentistry in the period between 2003 and 2005 ^13^. Level 1A had the smallest number of researchers but had the greatest quantity of publication and student advising. To achieve the 1A category, it is necessary to meet the requirements for Level 1B as well as to have contributed to the technological development of one's field in the country and have production with a socioeconomic effect, with activities that promote the development of products and processes available for society in the national and/or international markets ^5^.

The female sex predominated in the sample (66.7%) and on all research levels, which is similar to data reported for occupation therapy and education ^10^. However, this aspect is different in the fields of medicine ^14^ and biomedicine ^7^. Although women accounted for a greater number of researchers in the present investigation, men published more, on average, than women, which is in agreement with the greater participation of the male sex in scientific publications in journals of high prestige ^15^. In contrast, studies addressing funding for projects by the US National Institute of Dental and Craniofacial Research did not find significant differences between the sexes ^15^, reporting parity between the sexes of the first and last authors ^16^.

An analysis of the participation of women in articles published in 2006, 2011 and 2016 in periodicals with a high impact factor in the field of dentistry showed that women were represented little and that considerable inequality persists in scientific production between the sexes ^17^. In the world scenario, the participation of women in scientific publications in different fields of knowledge also suggests fewer publications compared to men ^15^ ^,^ ^17^. The traditional division of labor, by which women still perform a large part of household activities and provide care for the members of the family even in countries known for gender equality, may partially explain this gap ^18^.

The percentage of women in research increased to 57% in Brazil in the last 15 years, especially after the creation of the program Women and Science, which seeks gender equality in research ^5^ and the granting of maternity leave in cases of pregnancy or an adoption process (CONSELHO NACIONAL DE JUSTIÇA [National Justice Council], 1994). The International Association for Dental Research (IADR) has alerted the academic community with regards to this issue and creating opportunities for women who wish to progress in their careers ^19^. Besides the need for greater discussion regarding gender equality in publications, investigations that address the balance between personal and professional life are also necessary ^7^.

In the present study, the southeastern region of Brazil had the greatest number of research grant holders in pediatric dentistry, whereas none were found in the northern region of the country. Researchers from the southeastern region were found on all classification levels. Moreover, a greater number of publications and scientific collaborations among researchers from different Brazilian institutions were found in this region. VOSviewer revealed interinstitutional collaborations among CNPq productivity grant holders in the field of pediatric dentistry in different regions of Brazil and the nucleus of these collaborations were groups led by researchers of *Universidade Federal de Minas Gerais* and *Universidade de São Paulo* (USP) (São Paulo, Bauru and Ribeirão Preto campuses). The state of São Paulo has the greatest industrial production in Brazil as well as the highest gross domestic product. In 2016, wealth in this state accounted for 32.5% of the wealth of the entire country ^20^. The state of São Paulo concentrates renowned institutions and centers of scientific excellence, making it the largest center of research and development in the country ^21^. *Fundação de Amparo à Pesquisa do Estado de São Paulo* (FAPESP [State of São Paulo Research Assistance Foundation]) has received 1% of all revenue of the state monthly since 1989, ensuring the maintenance of fostering for research ^22^. The concentration of scientists as well as teaching and research institutes in this region is due to its greater economic development. Since the creation of financial incentives by the Health Ministry, Ministry of Science and Technology and state research support agencies, there has been concern for the decentralization of grants to other regions, as the southeastern region has historically concentrated research in the country ^23^. 

Most researchers in pediatric dentistry in the present study obtained their doctoral degrees by 2002 (median in the sample). However, researchers who obtained this title more recently predominated on Level 2. Nonetheless, no significant association was found between the year of obtainment of the doctoral degree and productivity grant level. The time of completing one's doctoral studies related to scientific production is one of the aspects analyzed for the awarding of a productivity grant ^24^. Researchers who completed their doctoral studies three years earlier are only accepted as Level 2 grant holders ^5^. This relationship may be influenced by scientific investment in the country and the quantity of research grants made available by CNPq in a given period of time. The literature reports that fewer productivity grant holders obtained the title of doctor in the last five years ^13^. Thus, a consolidated career and regular scientific production are determinants for obtaining a research grant. A large part of these grant holders has the title of full professor. In Brazil, nearly all funded research takes place at public universities ^23^ and the contractual relationship is important for researchers to be included in a human resources formation program ^24^.

More than half of the publications were JCR articles on all grant levels. The number of publications and JCR articles are parameters for the classification of CNPq grant levels ^7^ ^,^ ^24^. Another important aspect to the approval of CNPq researchers is work in an advising capacity. This aspect also enables links to other indirect financial incentives for the formation of these human resources ^24^. Generally, researchers on a higher level serve less in an advising capacity for scientific initiation and master's students. In the present investigation, however, researchers on higher levels served more in an advising capacity for such students. Moreover, more advisers of doctoral and post-doctoral students were found on Levels 1A, 1B and 1C than Level 2.

A study conducted with data on researchers from 195 countries from different regions of the world obtained total productivity and productivity in the last five years based on scores and citations of the i10 and h indices as well as citations in Google Scholar*.* Among the 10 thousand best researchers in Latin America, Brazil stood out due to its large number of researchers ^25^.

In the present study, total citations with and without self-citations were determined. Although often seen as inappropriate, self-citations cannot be disregarded. Authors who conducted studies in continuous series of cases need to cite their previous articles so that the reader can understand their research. Thus, self-citation does not constitute poor conduct, but rather a collaboration in a field of knowledge ^26^. In the present study, male researchers had proportionally more citations than female researchers.

The collection of information only from the CVs of the researchers on the Lattes platform was a methodological limitation of the present study due to differences in the filling out of data on the CVs analyzed. Moreover, scientific production may have been overestimated, as the same publications may have more than one researcher in pediatric dentistry. Despite these limitations, studies of this type are important to the scientific community and research fostering agencies. Moreover, the present investigation offers content that is relevant to the creation of programs that improve access to more researchers in dentistry and drive the development of the country.

Scientific production has increased in Brazil in recent years ^23^ but there is still a gap that needs to be filled regarding researchers and scientific collaborations in regions of the country beyond the southeastern region. There are also needs for financial incentives for researchers who recently obtained their doctoral degrees and more in-depth new investigations on gender differences in the obtainment of productivity grants. 

## Conclusions

CNPq grant holders with a background in pediatric dentistry are mainly female researchers from the southeastern region of the country. However, males have proportionally greater productivity.
